# Predictive value of OGTT parameters and clinical markers in gestational diabetes mellitus: a prospective randomized controlled trial from a tertiary center in Türkiye

**DOI:** 10.3389/fendo.2026.1793806

**Published:** 2026-03-04

**Authors:** Batuhan Turgay, Uğurcan Zorlu, Harun Kılıçkıran, Kayra Turgay, Gülşah Aynaoğlu Yıldız, Elif Gül Yapar Eyi, A. Seval Ozgu-Erdinc

**Affiliations:** 1 Department of Obstetrics and Gynecology, Ankara University Faculty of Medicine, Ankara, Türkiye. Reproductive Health Diagnosis Treatment Education Research and Application Center, Ankara University, Ankara, Türkiye; 2 Perinatology Unit, Ankara Bilkent City Hospital, Ankara, Türkiye; 3 Department of Obstetrics and Gynecology, Dörtyol State Hospital, Hatay, Türkiye; 4 Department of Pediatrics, Gazi University, Ankara, Türkiye; 5 Department of Obstetrics and Gynecology Perinatology Unit, Ankara University Faculty of Medicine, Ankara, Türkiye

**Keywords:** gestational diabetes mellitus, insulin therapy, oral glucose tolerance test, polyhydramnios, randomized controlled trial, risk stratification, ROC analysis

## Abstract

**Background:**

Gestational diabetes mellitus (GDM) remains a major obstetric concern, yet the optimal screening strategy and the prognostic value of oral glucose tolerance test (OGTT) parameters remain debated. We aimed to compare the diagnostic yield and clinical outcomes of a two-step OGTT strategy (50 g glucose challenge followed by 100 g OGTT) versus a one-step 75 g OGTT approach, and to evaluate the predictive performance of individual OGTT time points for pregnancy complications and treatment requirement.

**Methods:**

In this prospective randomized controlled trial, 1,439 pregnant women undergoing routine screening at 24–28 weeks of gestation were randomized to either a two-step OGTT strategy (n=719) or a one-step 75 g OGTT strategy (n=720). GDM was classified as diet-controlled or insulin-requiring. Maternal risk factors, obstetric outcomes, and neonatal outcomes were recorded. Receiver operating characteristic (ROC) analyses assessed the predictive ability of OGTT parameters for polyhydramnios and insulin requirement.

**Results:**

Overall GDM prevalence was 12.3%, including 8.4% diet-controlled and 3.9% insulin-requiring cases. The one-step strategy identified a numerically higher proportion of GDM without significant differences in maternal or neonatal outcomes compared with the two-step approach. Rates of polyhydramnios, hypertensive disorders, macrosomia, cesarean delivery, preterm birth, neonatal intensive care admission, small for gestational age (7.4%), and intrauterine growth restriction (4.2%) were comparable between groups. ROC analyses demonstrated that 2-hour OGTT values showed the strongest predictive performance for polyhydramnios (AUC up to 0.816) and insulin requirement (AUC up to 0.808), whereas the 50 g screening test showed only moderate discrimination.

**Conclusion:**

The one-step 75 g OGTT increases diagnostic labeling without improving short-term clinical outcomes. Post-load OGTT values—particularly 2-hour glucose levels—provide the most clinically meaningful prognostic information and may support a risk-stratified approach to GDM management rather than expansion of diagnostic thresholds alone.

## Introduction

Gestational diabetes mellitus (GDM) is a common metabolic complication of pregnancy and is associated with increased risks of polyhydramnios, hypertensive disorders, macrosomia, operative delivery, and long-term metabolic disease in both mothers and their offspring (1–3). Given its growing prevalence worldwide, accurate screening and clinically meaningful risk stratification remain central objectives of modern perinatal care (1, 2). Globally, GDM prevalence is estimated to range between approximately 7% and 15%, largely depending on diagnostic criteria and population characteristics (4, 5). In Türkiye, prospective cohort studies and randomized screening trials conducted in tertiary referral populations have reported prevalence rates broadly ranging between 8% and 17%, reflecting both regional metabolic risk profiles and variability in screening strategies (6, 7). These findings highlight a substantial and increasing national disease burden and emphasize the need for optimized risk-based screening approaches.

Currently, two main screening strategies are used in clinical practice: the two-step oral glucose tolerance test (OGTT) approach, consisting of a 50 g glucose challenge test followed by a diagnostic 100 g OGTT using Carpenter–Coustan criteria, and the one-step 75 g OGTT approach based on the International Association of Diabetes and Pregnancy Study Groups (IADPSG) recommendations (3, 8–10). Although the one-step strategy consistently increases the prevalence of GDM diagnosis, concerns persist regarding potential overdiagnosis and the lack of consistent evidence demonstrating improved maternal or neonatal outcomes (11–13). Randomized clinical studies comparing one-step and two-step screening strategies have suggested that broader diagnostic thresholds may increase case detection without proportionate reductions in clinically meaningful complications, contributing to ongoing debate regarding optimal screening policies in both global and regional populations (5).

Beyond screening strategy selection, increasing attention has focused on the prognostic significance of individual OGTT parameters. Previous studies suggest that post-load glucose values, particularly at later time points, may better reflect disease severity and predict adverse pregnancy outcomes or the need for insulin therapy (4, 14). However, most existing investigations have primarily evaluated diagnostic performance rather than prognostic stratification, and prospective randomized trials examining whether specific OGTT time points provide clinically actionable predictive information remain limited. This represents an important gap in the literature, especially within intermediate-risk populations such as those represented in Turkish tertiary-care cohorts (6, 7). Therefore, this study aimed to compare the diagnostic yield and clinical impact of the two-step and one-step screening approaches and to assess the predictive performance of OGTT parameters for pregnancy complications and treatment modality. We hypothesized that, irrespective of screening strategy, post-load OGTT values—particularly 2-hour glucose levels—would demonstrate superior predictive performance for clinically relevant outcomes compared with fasting measurements alone, supporting a more individualized risk-stratified framework for GDM management.

## Materials and methods

### Study design and setting

This investigation was conducted as a prospective, randomized controlled trial at Ankara Bilkent City Hospital, one of the largest tertiary referral centers in Türkiye, between 2022 and December 2023. The aim was to evaluate two different screening strategies for GDM and to assess their diagnostic yield and impact on maternal and neonatal outcomes. The study protocol was approved by the Institutional Ethics Committee (Approval No: E1-20-800). Written informed consent was obtained from all participants in accordance with the Declaration of Helsinki. NCT number is NCT04585204.

The study compared a two-step OGTT approach (50 g glucose challenge test followed by diagnostic 100 g OGTT using Carpenter–Coustan criteria) with a one-step 75 g OGTT approach based on International Association of Diabetes and Pregnancy Study Groups (IADPSG) recommendations.

Sample size estimation was based on detecting a clinically meaningful difference in GDM prevalence between screening strategies with an alpha level of 0.05 and a statistical power of 80%. The target sample size exceeded 1,400 participants to ensure adequate power for subgroup and ROC analyses.

Our randomized design allowed:

1. Comparison of GDM prevalence between the two-step OGTT and one-step 75 g OGTT approaches.
2. Assessment of whether the higher diagnosis rate translated into different maternal or neonatal outcomes.
3. Evaluation of the predictive performance of OGTT parameters for complications and treatment modality.

A total of 1,439 pregnant women presenting for routine GDM screening at 24–28 weeks of gestation were screened. Age ≥18 years, singleton pregnancy, no pregestational diabetes, and ability to provide consent were inclusion criteria. Pre-existing type 1 or type 2 diabetes, multiple gestation, chronic hepatic/renal/autoimmune disease, corticosteroid or glucose-altering drug use, refusal of consent, or incomplete OGTT data were exclusion criteria.

Participants were randomized in a 1:1 ratio using a computer-generated sequence prepared by an independent statistician. Allocation was concealed in sequential opaque envelopes. Participants and clinicians were not blinded due to the nature of glucose testing, but outcome assessors and statisticians remained blinded to allocation. Randomization was not stratified; however, maternal age and pre-pregnancy BMI were prespecified variables for adjusted and stratified analyses.

### Diagnostic protocols

Group A — Two-step OGTT approach (n=719):

- 50 g glucose challenge test (non-fasting, 1 h).
- If plasma glucose ≥140 mg/dL, a diagnostic 100 g OGTT was performed (fasting, 1-2–3 h).
- Carpenter–Coustan diagnostic thresholds were applied: fasting ≥95 mg/dL, 1 h ≥180 mg/dL, 2 h ≥155 mg/dL, and 3 h ≥140 mg/dL; ≥2 abnormal values were required for GDM diagnosis.

Group B — One-step 75 g OGTT approach (n=720):

- 75 g OGTT performed in the fasting state (0-1–2 h).
- IADPSG thresholds were used: fasting ≥92 mg/dL, 1 h ≥180 mg/dL, and 2 h ≥153 mg/dL; ≥1 abnormal value was sufficient for diagnosis.

Following diagnosis, women were classified as diet-controlled GDM (GDM-D), insulin-requiring GDM (GDM-I), or controls (normoglycemic). Women with incomplete OGTT results were excluded from per-protocol analysis but included in intention-to-treat analysis when possible. Missing categorical data (<5%) were excluded case-wise. Sensitivity analyses were conducted to evaluate the potential impact of missing data, and no significant differences were observed compared with the primary analysis.

### Outcomes

Primary outcomes:

1. GDM prevalence and distribution (diet vs insulin) between screening strategies.
2. Predictive value of OGTT parameters for polyhydramnios and treatment modality.

Secondary outcomes:

- Maternal outcomes: hypertensive disorders of pregnancy (including gestational hypertension, preeclampsia, and eclampsia), polyhydramnios, cesarean section, gestational weight gain.
- Neonatal outcomes: macrosomia, preterm delivery, NICU admission, low birth weight, small for gestational age (SGA), and intrauterine growth restriction (IUGR); outcomes with limited event numbers were reported descriptively.
- Risk factor associations, including pre-pregnancy body mass index (BMI), family history of diabetes mellitus, polycystic ovary syndrome (PCOS), and obstetric history.

### Statistical analysis

Analyses were conducted with SPSS version 25. Continuous variables were tested for normality using the Kolmogorov–Smirnov test. Parametric data are presented as mean ± standard deviation (SD), and non-parametric data as median [minimum–maximum]. Between-group comparisons were performed using the independent samples t-test or one-way ANOVA for parametric variables, and the Mann–Whitney U test or Kruskal–Wallis test for non-parametric variables. Categorical variables are expressed as frequencies and percentages and were compared using the Chi-square test or Fisher’s exact test, as appropriate.

**Figure 1:**
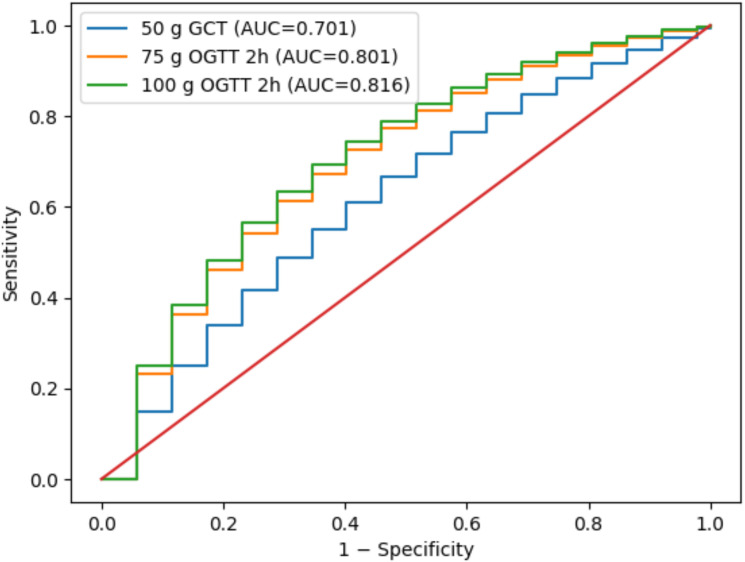
Hybrid ROC curves for prediction of polyhydramnios.

**Figure 2:**
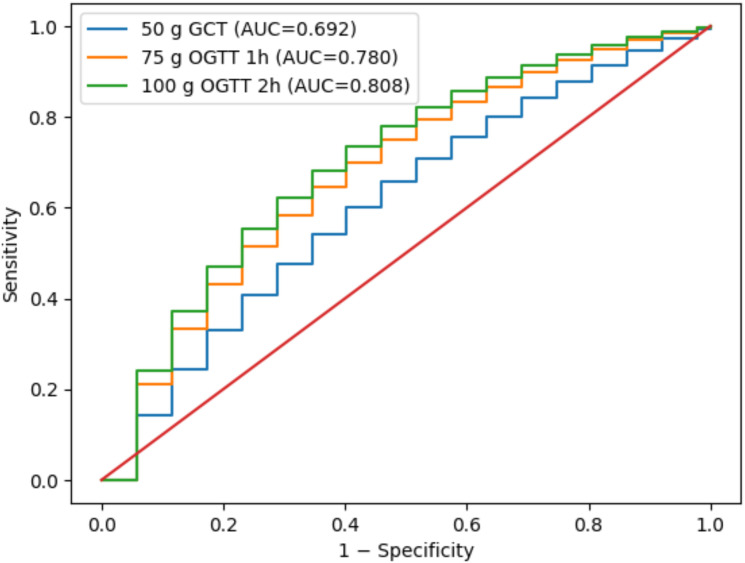
Stepwise ROC curves of OGTT parameters for prediction of insulin requirement.

Receiver operating characteristic (ROC) analyses were performed to evaluate the diagnostic performance of OGTT parameters for (i) prediction of polyhydramnios and (ii) differentiation between diet- and insulin-treated patients. AUC values with 95% confidence intervals, optimal cut-off values determined by the Youden Index, sensitivity, and specificity were calculated. ROC curves are presented graphically in **Figures 1**, **2**.

Multivariable logistic regression analyses were conducted to determine independent predictors of GDM diagnosis and insulin requirement, adjusting for maternal age, pre-pregnancy BMI, family history of diabetes, and PCOS. Additional stratified analyses were performed according to maternal age categories and BMI groups to evaluate potential effect modification.

All analyses were two-tailed, and a p value <0.05 was considered statistically significant.

## Results

### Baseline characteristics

A total of 1,439 women were randomized into two groups: 719 underwent the two-step OGTT approach (50 g screening followed by diagnostic 100 g OGTT) and 720 underwent the one-step 75 g OGTT approach. Of these, 177 (12.3%) were diagnosed with GDM, including 121 (8.4%) diet-controlled (GDM-D) and 56 (3.9%) insulin-requiring (GDM-I) cases, while 1,241 (87.7%) served as controls.

Baseline demographic characteristics are presented in **Table 1**. Maternal age was comparable across groups (Diet: 30.4 ± 3.3; Insulin: 30.6 ± 3.6; Control: 29.8 ± 3.5 years; p = 0.640). BMI values were also similar (Diet: 26.7 ± 2.4; Insulin: 26.9 ± 2.6; Control: 26.3 ± 2.3; p = 0.258). Gravida and parity distributions did not differ significantly among subgroups or between screening strategies. These findings confirm that randomization achieved well-balanced baseline demographics.

**Table 1:** Demographic characteristics of participants.

Characteristic	Diet (n=121)	Insulin (n=56)	Control (n=1262)	50 g group (n=719)	75 g group (n=720)	P-value
Age (mean ± SD)	30.4 ± 3.3	30.6 ± 3.6	29.8 ± 3.5	30.0 ± 3.4	30.2 ± 3.5	0.640
BMI (mean ± SD)	26.7 ± 2.4	26.9 ± 2.6	26.3 ± 2.3	26.5 ± 2.3	26.6 ± 2.4	0.258
Gravida (median [min–max])	2 (1–3)	2 (1–3)	2 (1–3)	2 (1–3)	2 (1–3)	0.721
Parity (median [min–max])	1 (0–2)	1 (0–2)	1 (0–2)	1 (0–2)	1 (0–2)	0.148

BMI, Body Mass Index; SD, Standard Deviation; min–max, minimum–maximum range.

p < 0.05 considered statistically significant.

Additional stratified analyses according to maternal age categories and pre-pregnancy BMI were performed, demonstrating no statistically significant interaction between screening strategy and these variables.

### Clinical and obstetric history

Clinical and obstetric characteristics are presented in **Table 2**. Women with GDM (both diet and insulin groups) had significantly higher prevalence of family history of diabetes, GDM in a first-degree relative, previous GDM, and previous infant >4,000 g (all p < 0.001). Other comorbidities (hypertension, history of embolism, IVF conception, prior polyhydramnios) were not significantly different between groups (all p > 0.05), confirming comparable baseline obstetric risk profiles between screening strategies.

**Table 2:** Clinical and obstetric characteristics related to GDM.

Characteristic	Diet (%)	Insulin (%)	Control (%)	50 g group (%)	75 g group (%)	P-value
Presence of comorbidities	28.9	29.4	20.1	23.5	25.3	0.220
History of IUFD	5.8	8.7	5.2	5.5	6.1	0.331
IVF/IUI conception	12.4	15.9	10.8	11.1	11.8	0.287
Hypertension	7.4	9.1	4.6	6.1	6.3	0.185
History of embolism	0.8	3.7	0.7	1.5	1.9	0.273
Family history of DM	25.6	26.5	10.1	14.8	15.6	<0.001
GDM in sister	14.9	20.4	2.8	7.1	7.8	<0.001
Previous pregnancy with GDM	13.2	22.1	2.1	6.5	7.0	<0.001
Previous infant >4 kg	18.2	21.5	8.5	12.2	13.1	<0.001
Previous polyhydramnios	9.9	12.3	4.7	6.4	7.2	0.195

DM, Diabetes Mellitus; GDM, Gestational Diabetes Mellitus; IVF/IUI, *In Vitro* Fertilization/Intrauterine Insemination; IUFD, Intrauterine Fetal Death.

p < 0.05 considered statistically significant.

### Lifestyle and pregnancy factors

Lifestyle characteristics are summarized in **Table 3**. Rates of regular exercise (25–27%), smoking (8–11%), alcohol consumption (2–4%), and vitamin supplementation (87–90%) were similar across groups (all p > 0.05). However, PCOS prevalence was higher among women with GDM (Diet: 17.8%; Insulin: 18.2%; Control: 7.9%; p < 0.001). Gestational weight gain was significantly higher in the insulin group (13.0 ± 3.8 kg) compared to diet-treated (11.7 ± 3.1 kg) and control women (12.0 ± 2.9 kg; p = 0.018). Current pregnancy polyhydramnios was numerically higher in the insulin group but did not reach statistical significance.

**Table 3:** Lifestyle and clinical factors during pregnancy.

Characteristic	Diet (%)	Insulin (%)	Control (%)	50 g group (%)	75 g group (%)	P-value
Regular exercise	25.2	23.0	27.0	25.8	26.2	0.685
Presence of PCOS	17.8	18.2	7.9	11.1	11.5	<0.001
Smoking	9.9	8.7	10.7	9.8	9.9	0.870
Alcohol consumption	2.4	2.0	4.1	3.5	3.1	0.520
Vitamin supplementation	89.5	89.0	87.3	88.4	88.2	0.712
Polyhydramnios (current)	7.6	10.9	5.3	6.0	6.5	0.210
Gestational weight gain (kg, mean ± SD)	11.7 ± 3.1	13.0 ± 3.8	12.0 ± 2.9	11.9 ± 3.1	12.1 ± 3.2	0.018

PCOS, Polycystic Ovary Syndrome; SD, Standard Deviation.

p < 0.05 considered statistically significant.

### Diagnostic yield of screening protocols

A total of 177 women (12.3%) were diagnosed with gestational diabetes mellitus. Although the prevalence of GDM was numerically higher in the one-step 75 g OGTT approach compared with the two-step OGTT approach, this difference did not reach statistical significance (12.9% vs 11.7%, p = .318) (**Table 4**).

**Table 4:** Diagnostic yield by randomization group.

Group	Total N	GDM, n (%)	Diet-GDM, n (%)	Insulin-GDM, n (%)	Control, n (%)
50 g two-step group	719	84 (11.7)	56 (7.8)	28 (3.9)	635 (88.3)
75 g one-step group	720	93 (12.9)	65 (9.0)	28 (3.9)	627 (87.1)

Values are presented as *n* (%). Group comparisons were performed using the chi-square test. p = .318.

### Maternal and neonatal outcomes

Maternal and neonatal outcomes are summarized in **Table 5**. No significant differences were observed between screening strategies in polyhydramnios (6.1% vs 6.5%), hypertensive disorders of pregnancy (5.9% vs 6.1%), macrosomia (>4000 g: 9.0% vs 9.4%), preterm birth (<37 weeks: 8.1% vs 8.3%), NICU admission (10.2% vs 10.5%), or cesarean delivery (38.5% vs 39.1%) (all p > 0.05).

**Table 5:** Maternal and neonatal outcomes by randomization group.

Outcome	50 g group (%)	75 g group (%)	P-value
Polyhydramnios	6.1	6.5	0.742
Hypertensive disorders	5.9	6.1	0.801
Macrosomia (>4000 g)	9.0	9.4	0.851
Preterm birth (<37 wks)	8.1	8.3	0.877
NICU admission	10.2	10.5	0.903
Cesarean section	38.5	39.1	0.715

NICU, Neonatal Intensive Care Unit; wks, weeks of gestation.

No significant differences were observed (all p > 0.05).

Rates of fetal growth–related outcomes remained low overall. The prevalence of small for gestational age (SGA) was 7.4% and intrauterine growth restriction (IUGR) was 4.2%, values consistent with expected ranges reported in comparable obstetric populations. No statistically or clinically meaningful differences were observed between the two-step OGTT and one-step 75 g OGTT screening strategies for these outcomes (all p > 0.05). These findings indicate that the increased diagnostic rate observed with the one-step approach did not translate into improved neonatal growth-related outcomes.

### Predictive performance of OGTT parameters

The predictive ability of OGTT parameters for polyhydramnios is presented in **Table 6** and illustrated in **Figure 1**. The strongest predictors were the 100 g OGTT 2 h value (AUC = 0.816) and the 75 g OGTT 2 h value (AUC = 0.801), both demonstrating high discriminatory power. The 75 g 1 h value also showed good predictive performance (AUC = 0.782), whereas the 50 g screening test demonstrated only moderate predictive ability (AUC = 0.701).

**Table 6:** Predictive performance of OGTT parameters for polyhydramnios.

Test	AUC	95% CI	Cut-off (Youden)	Sensitivity	Specificity
50 g OGTT	0.701	0.639–0.767	142 mg/dL	0.658	0.689
75 g OGTT – 0h	0.664	0.592–0.731	92 mg/dL	0.623	0.674
75 g OGTT – 1h	0.782	0.724–0.835	184 mg/dL	0.740	0.757
75 g OGTT – 2h	0.801	0.748–0.855	158 mg/dL	0.769	0.776
100 g OGTT – 0h	0.677	0.615–0.735	96 mg/dL	0.642	0.687
100 g OGTT – 1h	0.789	0.734–0.842	185 mg/dL	0.747	0.764
100 g OGTT – 2h	0.816	0.768–0.864	160 mg/dL	0.782	0.780
100 g OGTT – 3h	0.773	0.716–0.831	143 mg/dL	0.732	0.753

OGTT, Oral Glucose Tolerance Test; AUC, Area Under Curve; CI, Confidence Interval; Youden, Optimal cut-off point determined by Youden Index.

ROC analysis for polyhydramnios prediction.

For differentiating diet-controlled versus insulin-requiring GDM (**Table 7**, **Figure 2**), the 100 g OGTT 2 h value again performed best (AUC = 0.808). The 75 g OGTT 1 h and 2 h values also demonstrated strong discrimination (AUC range: 0.765–0.780), while the 50 g challenge test showed lower performance (AUC = 0.692). These findings highlight that post-load OGTT parameters, particularly 2-hour glucose values, provide the most clinically useful predictive information for both complications and treatment stratification.

**Table 7:** ROC analysis of OGTT parameters for distinguishing GDM treatment subgroups (Diet-Controlled vs. Insulin-Requiring).

Test	AUC	95% CI	Cut-off (Youden)	Sensitivity	Specificity
50 g OGTT	0.692	0.631–0.750	147 mg/dL	0.700	0.662
75 g OGTT – 0h	0.751	0.691–0.804	95 mg/dL	0.728	0.707
75 g OGTT – 1h	0.780	0.723–0.832	186 mg/dL	0.745	0.750
75 g OGTT – 2h	0.765	0.706–0.823	162 mg/dL	0.713	0.760
100 g OGTT – 0h	0.762	0.703–0.822	97 mg/dL	0.734	0.742
100 g OGTT – 1h	0.787	0.732–0.839	188 mg/dL	0.758	0.766
100 g OGTT – 2h	0.808	0.756–0.859	161 mg/dL	0.780	0.770
100 g OGTT – 3h	0.770	0.710–0.827	143 mg/dL	0.727	0.759

OGTT, Oral Glucose Tolerance Test; AUC, Area Under Curve; CI, Confidence Interval; Youden, Optimal cut-off point determined by Youden Index.

ROC analysis to differentiate diet-controlled vs insulin-requiring GDM.

## Discussion

GDM remains a major obstetric challenge due to its well-established associations with adverse maternal and neonatal outcomes and its long-term metabolic implications. Screening strategies aim to balance early identification of at-risk pregnancies with avoidance of unnecessary medicalization. In this prospective randomized controlled trial, we compared two widely used screening approaches and evaluated the prognostic value of individual OGTT parameters within a tertiary-care population.

Although the one-step 75 g OGTT strategy has been increasingly adopted in clinical practice, concerns remain regarding whether expanded diagnostic thresholds translate into meaningful improvements in outcomes. Our findings demonstrate that despite a numerically higher rate of GDM diagnosis with the one-step approach, maternal and neonatal outcomes were largely comparable between screening strategies. These results are consistent with prior randomized and observational data suggesting that broader diagnostic criteria may increase case detection without proportional clinical benefit.

A key strength of this study lies in evaluating OGTT parameters as prognostic markers rather than solely diagnostic thresholds. We observed that post-load glucose values, particularly 2-hour measurements, provided the strongest predictive performance for polyhydramnios and for distinguishing diet-controlled from insulin-requiring GDM. Previous investigations have similarly reported that elevated OGTT values are closely associated with increased likelihood of pharmacologic treatment and metabolic severity (15). Furthermore, evidence from randomized trials evaluating treatment effects in mild GDM indicates that glucose patterns across the OGTT may better reflect clinical risk than fasting glucose values alone (16). Together, these findings support the concept that dynamic glycemic responses provide clinically actionable information beyond binary diagnostic cut-offs.

From a biological standpoint, elevated late OGTT values may reflect impaired peripheral glucose utilization and progressive insulin resistance mediated by placental hormones. Studies examining selective screening strategies have demonstrated that post-challenge glucose levels correlate with metabolic burden and adverse outcomes even within intermediate-risk populations (17). Similarly, early pregnancy metabolic and inflammatory markers have been linked to subsequent GDM development, underscoring the multifactorial nature of glucose dysregulation during pregnancy (18, 19). When interpreted alongside our findings, these data support a multidimensional risk-stratified approach that integrates OGTT profiles with additional metabolic indicators.

Importantly, although the one-step strategy identified more women with milder glycemic abnormalities, this increase did not result in significant differences in clinically relevant outcomes, including polyhydramnios, hypertensive disorders, macrosomia, small for gestational age, intrauterine growth restriction, or neonatal intensive care unit admission. These observations suggest that expanding diagnostic thresholds alone may increase healthcare utilization without clear clinical benefit, highlighting the need for individualized risk assessment rather than universal diagnostic expansion.

To our knowledge, this study represents one of the few prospective randomized investigations conducted in a Turkish tertiary-care population that simultaneously compares screening strategies while evaluating the prognostic performance of individual OGTT time points. Unlike prior studies primarily focused on diagnostic prevalence, our analysis integrates ROC-based predictive modeling with clinically meaningful maternal and neonatal outcomes, providing a more comprehensive framework for risk-stratified GDM management. This dual diagnostic–prognostic approach may help refine screening policies in populations with intermediate metabolic risk profiles.

The strengths of this trial include its randomized design, large sample size, standardized screening protocols, and comprehensive assessment of maternal and neonatal outcomes. Nevertheless, several limitations should be acknowledged. The single-center design may limit generalizability, and long-term metabolic follow-up of mothers and offspring was not evaluated.

From a clinical and public health perspective, our findings suggest that individualized interpretation of OGTT profiles—particularly post-load glucose patterns—may offer greater value than expanding diagnostic thresholds alone. Future multicenter studies integrating OGTT-derived metrics with emerging metabolic and inflammatory biomarkers may further enhance personalized screening and management strategies for gestational diabetes mellitus.

## Conclusion

While the one-step 75 g OGTT strategy may identify a numerically greater proportion of women with GDM, this does not necessarily translate into improved clinical outcomes. Individual OGTT parameters—particularly 2-hour post-load glucose values—appear to offer greater prognostic utility for complications and treatment requirements. Future research should focus on validating OGTT-based risk stratification models and integrating them with emerging biomarkers to optimize personalized management of GDM.

## Data Availability

The raw data supporting the conclusions of this article will be made available by the authors, upon request, without undue reservation.
